# Lactate-Protected Hypoglycemia (LPH)

**DOI:** 10.3389/fnins.2020.00920

**Published:** 2020-09-03

**Authors:** Matthew L. Goodwin, L. Bruce Gladden, Maarten W. N. Nijsten

**Affiliations:** ^1^Department of Orthopedic Surgery, Washington University in St. Louis, St. Louis, MO, United States; ^2^School of Kinesiology, Auburn University, Auburn, AL, United States; ^3^Critical Care Department, University Medical Center Groningen, Groningen, Netherlands

**Keywords:** lactate, hypoglycemia, shuttle, hyperlactatemia, monocarboxylate transporter, lactate dehydrogenase

## Abstract

Here, we provide an overview of the concept of a lactate-protected hypoglycemia (“LPH”), originally proposed as lowering glucose while simultaneously increasing lactate concentration as a method by which tumors might be targeted. Central to this hypothesis is that lactate can act as a critical salvage fuel for the central nervous system, allowing for wide perturbations in whole body and central nervous system glucose concentrations. Further, many tumors exhibit “the Warburg” effect, consuming glucose and producing and exporting lactate despite adequate oxygenation. While some recent data have provided evidence for a “reverse-Warburg,” where some tumors may preferentially consume lactate, many of these experimental methods rely on a significant elevation in lactate in the tumor microenvironment. To date it remains unclear how various tumors behave *in vivo*, and how they might respond to perturbations in lactate and glucose concentrations or transport inhibition. By exploiting and targeting lactate transport and metabolism in tumors (with a combination of changes in lactate and glucose concentrations, transport inhibitors, etc.), we can begin developing novel methods for targeting otherwise difficult to treat pathologies in the brain and spinal cord. Here we discuss evidence both experimental and observational, and provide direction for next steps in developing therapies based on these concepts.

## Introduction: Lactate as Central to Metabolism

Beginning with its discovery in 1780, lactate was once thought of as a dead-end waste product, formed in times of poor perfusion or hypoxia (Ferguson et al., 2018). This view dominated for much of the ensuing 200 years, until the 1980’s, when, on the basis of studies on both animals and humans, George Brooks proposed the cell-to-cell lactate shuttle hypothesis (Brooks, 2002, 2018; Gladden, 2004). Those studies ranged from isolated organs to intact humans, employing techniques such as muscle biopsies, arteriovenous differences combined with tissue blood flow, and radioactive tracer methods. Evidence supporting this hypothesis has continued to accumulate and it is now universally accepted as biological theory (Brooks, 2002, 2018; Gladden, 2004; Ferguson et al., 2018). The cell-cell shuttle is the mechanism by which whole body lactate metabolism is coordinated. Rather than a waste product formed during hypoxia, lactate is a dynamic intermediate that is constantly produced and consumed, exported from some tissues and taken up by others to be oxidized as fuel or stored as glycogen. While oxygen is required for oxidative phosphorylation, the pO_2_ experienced *in vivo* is almost never low enough to limit cytochrome turnover significantly below its maximum rate (Richardson et al., 1995; Heino et al., 1997). Contrary to the original teaching and dogma, lactate is being produced, circulated, and consumed in a near-independent manner from oxygen.

We now know that skeletal muscle serves as a fuel reservoir, with glycogen stores that can be broken down to lactate in response to catecholamines binding β-adrenergic receptors on the muscle cell membrane (Daniel et al., 1978; Gore et al., 1991; Garcia-Alvarez et al., 2014; Goodwin et al., 2019). Lactate can then passively diffuse down its concentration gradient out of the cell and into other tissues via monocarboxylate transporters (MCTs), where it can be used as a fuel (Halestrap and Price, 1999). In times of whole animal stress (i.e., fight-or-flight, trauma, burns, traumatic brain injury), this mechanism allows rapid mobilization of fuel at little cost (Gore et al., 1991; Garcia-Alvarez et al., 2014; Brooks and Martin, 2015). The enzyme lactate dehydrogenase (LDH) catalyzes the near-equilibrium pyruvate to lactate reaction. Lactate dehydrogenase is present at a higher activity than any of the other enzymes in either glycolysis or oxidative metabolism. As a result, lactate is constantly being formed and consumed almost instantaneously (Goodwin et al., 2015; Rogatzki et al., 2015; Bak and Schousboe, 2017). With its large reservoir in the form of glycogen, movement down concentration gradients without the need for energy input, and rapid conversion to pyruvate once inside a cell, lactate is an excellent fuel source for most tissues of the body; the central nervous system is no exception (Gladden, 2000; Brooks and Martin, 2015; Brooks, 2018; Ferguson et al., 2018).

Pellerin and Magistretti (1994) introduced the original astrocyte-neuron lactate shuttle (ANLS), proposing that astrocytes take up glucose and then release lactate for use by nearby neurons. In this model MCT1s and MCT4s have been proposed as responsible for exporting lactate from astrocytes, while MCT2s have been proposed as importing lactate into neurons (Pellerin and Magistretti, 2012). While MCTs are bidirectional in nature, studies on their distribution have proven their expression to be tissue-specific: MCT1s are expressed in astrocytes, oligodendrocytes, endothelial cells, and many other tissues throughout the body, MCT4s are expressed in astrocytes but not neurons (as well as in muscle cells and a few other places throughout the body), and MCT2s are found predominantly in neurons. While a comprehensive review of MCTs is beyond the scope of this paper, it should be noted that the affinity of each MCT for lactate varies markedly and likely contributes to their tissue-specific expression. For example, MCT2 has a high affinity for lactate (Km ∼ 0.7 mM) and its expression has proven to be inducible, such that a rapid increase in expression can allow for rapid changes in lactate transport (Pellerin and Magistretti, 2012). MCT1 and MCT4, in contrast, have a lower affinity for lactate (Kms of ∼3.5 and 34 mM, respectively). While affinity for lactate and thus responsiveness varies, care must be taken not to confuse this with directionality, which is ultimately determined by lactate concentration. Depending on the metabolic state, for example, MCT1s may function as lactate importers or exporters. While the ANLS concept and the experiments supporting it have been debated, the importance of lactate within the CNS is clear. Lactate, whether formed by astrocytes, or by crossing the blood-brain barrier itself (Pardridge, 1986), is clearly a major player in the central nervous system in both health and disease (Brooks, 2018). To date, data continue to accrue in favor of lactate as a primary metabolite at the center of metabolism (Hensley et al., 2016; Faubert et al., 2017; Harjes, 2017; Hui et al., 2017; Brooks, 2018).

Recently, studies on lactate in the tumor microenvironment have shed considerable light on lactate’s role in metabolism. Studies on tumor metabolism have dated back to the 1920’s, when it was reported that tumors in solution acidified the surrounding environment and experienced an elevated [lactate] in response to the addition of glucose (Warburg and Minami, 1923; Ferguson et al., 2018). The Cori and Cori (1925) reported that venous blood draining a sarcoma-bearing wing was higher in [lactate] [and lower in (glucose)] than the contralateral wing, while Otto Warburg observed that lactate production in rat hepatoma slices was on the order of ∼70 times that seen in normal tissue, regardless of O_2_ availability (Otto, 2016). When Warburg examined the inflow and outflow of tumors in rats, he found that the artery feeding the tumor always had a higher [glucose] and lower [lactate] then the vein draining it (Warburg et al., 1927). This glucose-avid, lactate-producing phenotype has since been dubbed the “Warburg effect,” and has been demonstrated across a wide variety of tumor types, forming the basis of the extra-ordinary ability of the FDG-PET scan to detect most types of solid cancers.

With the introduction of the cell-cell lactate shuttle in the 1980s, a new understanding of lactate metabolism emerged. However, it was not until 2008, with the publication suggesting there was lactate shuttling within tumors from Sonveaux et al. (2008), that lactate made its appearance on center stage in oncology. In his commentary summarizing this work, Gregg Semenza, himself the discoverer of HIF-1α and Nobel Laureate, paid homage to the work done in muscle physiology on lactate shuttling, “Was there any precedent that should have alerted us to the existence of this symbiotic relationship between aerobic and hypoxic cancer cells? Of course; the well-known recycling of lactate in exercising muscle (Semenza, 2008).” Sonveaux et al. (2008) demonstrated that some tumor cells seem better suited for oxidative metabolism and the use of lactate as a fuel than are others. Studying human cervix squamous carcinoma cells (SiHa) and human colorectal adenocarcinoma cells (WiDr), they demonstrated that SiHa cells were oxidative and would readily use lactate as fuel, whereas WiDr cells were more glycolytic, consuming glucose and producing lactate in a Warburg-type manner. MCT1 inhibition (or silencing) blocked lactate-fueled respiration of the SiHa cells. These same researchers slowed tumor growth in Lewis lung carcinoma (LLc) and WiDr mouse models by injecting the MCT inhibitor a-cyano-4-hydroxycinnamate (CHC). However, this treatment was unsuccessful in slowing growth in transplantable liver tumor cells (TLT), which have very little expression of MCT1. These authors posited that cells at the hypoxic core of a tumor may produce lactate by necessity, while more peripheral cells with ample O_2_ supply could then take up that lactate and use it as a fuel. However, while both WiDr and SiHa cells used lactate to some degree when no glucose was available, no definitive data demonstrated that cancer cells of the same type might shuttle lactate within one tumor microenvironment. Further, the lack of control cells in these studies makes comparison to “normal” metabolism difficult. Unfortunately, how to use these and other data to progress to the next step in a translational model is unclear; the experimental conditions are too far removed from *in vivo* conditions (Muir et al., 2018). For example, human cervix squamous carcinoma, characterized by Sonveaux et al. (2008) as oxidative and a “lactate consumer” (or “Reverse Warburg”) is a glucose-consuming, PET-positive tumor *in vivo* (i.e., lactate-producing or “Warburg”) (Walenta et al., 2000).

Using a “lactate-protected hypoglycemia (LPH)” as a strategy to target glucose-avid, lactate-producing tumors was first described in 2008 by Nijsten and Van Dam (2009). In this proposal the authors posit that the Warburg behavior of tumors might be targeted by inducing a deep hypoglycemia while providing lactate as a salvage fuel for non-tumorous tissues. Since this was proposed, however, two areas of research have suggested this may be quite difficult to implement as a therapeutic treatment on its own: (1) lactate’s emerging role as a signaling molecule in tumorigeness and (2) tumors that appear to demonstrate a “reverse Warburg” phenotype, consuming lactate.

**FIGURE 1:**
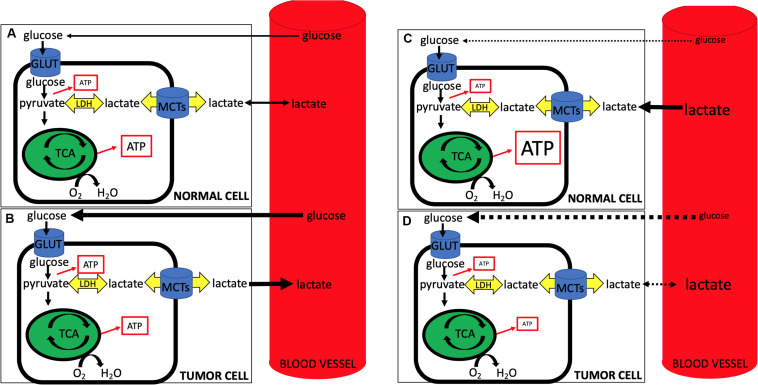
During normal conditions **(A,B)**, circulating glucose is taken up by normal **(A)** and tumor **(B)** cells, with tumor cells being more glucose-avid (represented by the thicker black line denoting glucose from blood to the cell) and exporting more lactate (represented by the thicker black line denoting lactate from cell to blood). Note the relative increase in ATP production from glycolysis in tumor cells and the concomitant slightly less ATP from oxidative phosphorylation (represented by size of ATP in box). During lactate-protected hypoglycemia (LPH) **(C,D)**, circulating glucose is dramatically lowered and glucose uptake into both normal **(C)** and tumor **(D)** cells is impaired (dotted lines from blood to cell glucose). At the same time hyperlactatemia serves as a salvage fuel for normal cells, while tumor cells, accustomed to lactate export, have a less robust ability to import and use lactate. Note decreased ATP production via glycolysis from both cells with an increase in oxidative phosphorylation ATP production from lactate import in normal cells and an overall impaired ATP production from tumor cells. Note that LPH alone, while elegant in its use of lactate as a salvage fuel for the CNS, may prove to be problematic as tumor cells may adjust and begin taking up lactate when (1) glucose becomes limiting, and/or (2) lactate becomes abundant. Further, MCT expression profiles in tumors often differ when compared to non-tumor tissues, suggesting that the use of LPH in conjunction with selective targeting of tumor MCTs may be more effective than LPH alone.

First, lactate’s role has expanded beyond that of a metabolite – it is now known to function as a signaling molecule as well (Errea et al., 2016; Brown et al., 2020). Although beyond the scope of this review, one of the most intriguing roles lactate plays is as the primary ligand for the hydroxycarboxylic acid receptor 1 (HCAR1), also known as GPR81. This G-protein coupled receptor appears to promote tumorigenesis in both an autocrine and paracrine fashion (Brown et al., 2020; Hu et al., 2020). LPH as a strategy to target tumors becomes increasingly more challenging if lactate itself is a ligand that binds to stimulate oncogenic pathways. For example, in the case of GPR81, lactate binding and the resultant tumorigenesis is independent of transport via MCTs. Currently work is ongoing in this area. Second, recent studies demonstrating lactate-consuming behavior of tumor cells seems to conflict with previous data. For example, how do we reconcile recent studies that demonstrate a reverse-Warburg phenotype in tumors that clinically are glucose-consuming, PET-positive, Warburg-type cells? And where does this leave our LPH hypothesis, whereby inducing a whole-animal hypoglycemia may starve tumor cells, while providing lactate in the form of hyperlactatemia may act as a salvage fuel for non-tumor tissues (Figure 1)?

Many recent studies that suggest lactate is a preferred fuel source of tumor cells fail to recognize the importance of the extracellular lactate concentration as a determinant of the direction of lactate diffusion (i.e., into or out of the cell). MCTs are bidirectional transporters and can function both to import and export lactate, albeit with a differing *K*_*m*_ and thus differing responsiveness to concentration changes (Halestrap and Price, 1999; Ferguson et al., 2018; Glancy et al., 2020). Bonuccelli et al. (2014) demonstrated that osteosarcoma cells consume lactate, whereas Sonveaux et al. (2008) posited the same with cervical cancer cells, and still others have made similar claims with a variety of other cancer cells and models (Sanchez et al., 2013; Pértega-Gomes and Baltazar, 2014; Faubert et al., 2017; Harjes, 2017). However, these experiments typically involve use of supraphysiologic concentrations of lactate (e.g., ∼10 mM). In mice, an alveolar soft parts sarcoma (ASPS) model demonstrated that lactate drove tumorigenesis, acting as a fuel and driving HIF1α (Goodwin et al., 2014). Tumors in this model, as noted, *only* formed in areas of high lactate concentration (all formed in the cranial vault, where lactate concentration was on the order of 6–9 mM). Work by Pagliassotti and Donovan (1990) in muscle demonstrated long ago that even the most glycolytic, lactate-producing muscles will revert to taking up lactate as the systemic lactate perfusing them rises above 4 mM. In the ASPS mouse, a high lactate concentration allowed for tumor initiation and growth; augmentation with daily lactate injections further increased vascularity and markers of proliferation (Ki67) within these tumors (Goodwin et al., 2014). All of these environments (ASPS mice and isolated cells) contain a higher extracellular lactate concentration than what is found in the human extracellular milieu of the CNS (∼2.5 mM) (Turner and Adamson, 2011). This small factor is critical when determining if and when tumors produce or consume lactate. In high-lactate environments, almost all tissues will take up lactate. The results – that isolated cells in 10 mM lactate consume lactate, or ASPS mouse tumor cells in 6–9 mM take up lactate – should not surprise us.

Overall, it is likely that many tumor cells are glucose-avid, PET-positive Warburg cells. However, when glucose becomes limiting or lactate becomes abundant, both the CNS and many tumors can likely readily use lactate as a fuel. LPH, while elegant in its use of lactate as a salvage fuel for the CNS, may prove to be problematic as tumor cells adjust and begin taking up lactate when (1) glucose becomes limiting, and/or (2) lactate becomes abundant. Further, lactate itself may act as a signaling molecule, further impeding efforts to use it as a salvage fuel. Given this, the use of LPH in conjunction with selective targeting of tumor cell lactate transport (MCTs) seems more likely to be efficacious than LPH alone.

To summarize, lactate is a valuable metabolite that is constantly shuttling between tissues, coordinating whole-body and CNS metabolism. While isolated studies have suggested some cancer cells may consume lactate rather than produce it, this is likely due in large part to the experimental designs used, where lactate is ∼10 times normal. In attempts to use LPH as a strategy to target tumors *in vivo*, care must be taken to ensure either (1) lactate transport into tumor cells is specifically inhibited, or (2) tumor-perfusing blood lactate concentrations are not high enough to induce lactate uptake by a tumor cell that otherwise would prefer lactate output. The efficacy of LPH in targeting tumor cell metabolism, alone or as an adjuvant, is yet to be determined in humans.

## Data Supporting LPH *in vitro*

Both human case reports and animal studies provide indirect evidence that lactate can serve as a salvage fuel during times of severe hypoglycemia (Gladden et al., 2011; Oldenbeuving et al., 2014; Ferguson et al., 2018; Goodwin et al., 2019; Glancy et al., 2020). More direct evidence is littered throughout the literature and much of the detail is beyond the scope of this brief commentary, as it is now well-accepted within the framework of the cell-cell lactate shuttle and the astrocyte-neuron lactate shuttle (Brooks, 2018; Glancy et al., 2020). In Avital Schurr’s seminal work in *Science* in 1988 (Schurr et al., 1988), rat hippocampus slices were isolated and membrane potentials measured. When glucose was removed, tissue slices rapidly lost membrane potential; then when lactate was added, the membrane potential quickly recovered. This concept has been repeated in a variety of cellular experiments, demonstrating lactate’s ability to diffuse into cells via MCTs, where it can then be utilized as a fuel (Brooks, 2002, 2018). Now, advances like the Seahorse assay purport to quantify cell metabolism via extracellular acidification rate (ECAR) as well as oxygen consumption rate (OCR) (van der Windt et al., 2017). While limits to the Seahorse assay (and others like it) exist, when used in conjunction with studies examining MCT expression, lactate and glucose concentrations, and whole genome transcriptome changes, any given tissue’s reliance on lactate as a fuel can be readily quantified and compared to other tissues.

## Data for LPH *in vivo*

Nijsten and van Dam (2009) proposed the framework for LPH based on a variety of studies both *in vitro* and *in vivo*; the data support lactate as a potent fuel source for the CNS. Oldenbeuving et al. (2014) reported the case of a patient who presented to the Emergency Department ambulatory and conversant despite a glucose level of only 0.7 mM (∼12.6 mg/dL). Normally this level of hypoglycemia is associated with coma or even death. In this case report the patient was in hepatic failure and lacked the ability to synthesize new glucose at the liver via gluconeogenesis. However, his arterial lactate was 25 mM. Given what is known about the central nervous system’s reliance on lactate, this case report provides circumstantial evidence for lactate acting as a CNS salvage fuel during hypoglycemia (for full discussion see Faubert et al., 2017). Other indirect evidence for lactate as a salvage fuel exists, including neonatal lactate concentrations on the order of 6–9 mM before the liver is mature enough for gluconeogenesis (Medina, 1985; Ohki et al., 1999).

**FIGURE 2:**
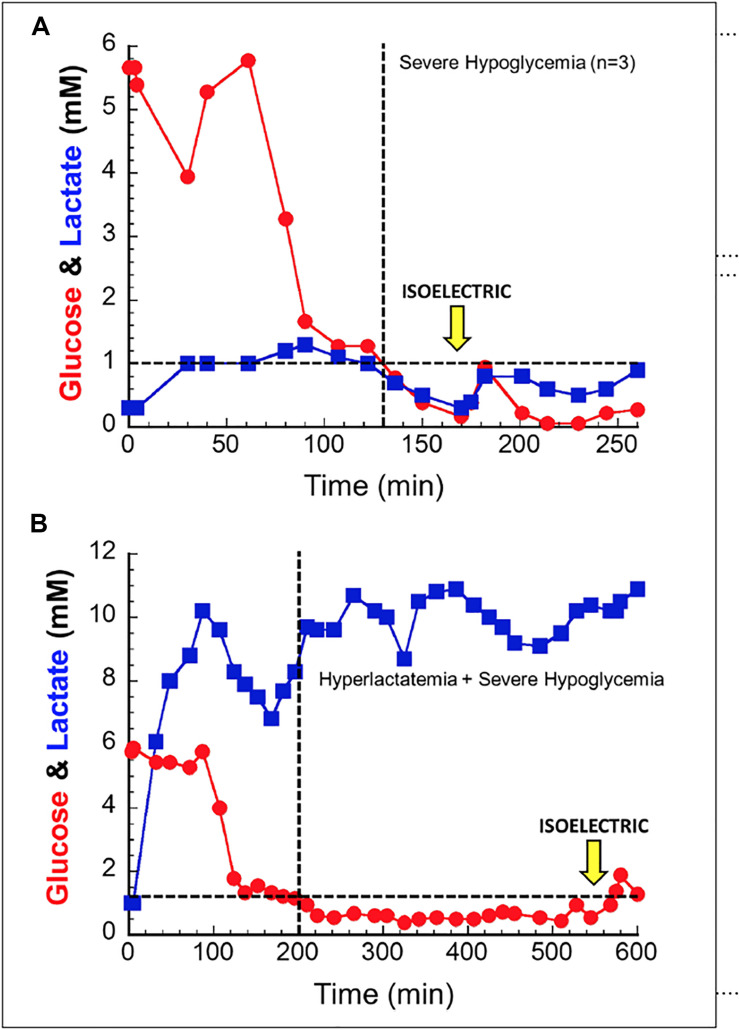
In anesthetized canines, hypoglycemia without a salvage fuel is not well tolerated **(A)**; isoelectric brain death is seen around 30 min after hypoglycemia. When lactate is provided as a salvage fuel **(B)**, brain activity is maintained for 6–8 h after hypoglycemia induction. Note the difference in scale of the *X* and *Y* axes of the two figures. Used with permission from Ferguson et al. (2018).

In order to better understand lactate’s ability to serve as a salvage fuel during extreme hypoglycemia, we performed a series of trials in anesthetized dogs in which severe hypoglycemia was induced during hyperlactatemia (Ferguson et al., 2018). In these experiments animals were infused with sodium lactate for up to 10 h while acid base balance was controlled. The primary endpoint was brain electrical activity (EEG). After induction of hyperlactatemia, hypoglycemia was induced through a combination of insulin, -β-blockade, biguanides, and ethanol. During trials of deep hypoglycemia without lactate infusion, brain death ensued within ≈30 min. When lactate was present as a salvage fuel (∼10 mM), animals lived for up to ∼6–8 h (Figure 2).

While early data for LPH as a therapy are promising, challenges remain. Many tissues of the central nervous system are capable of utilizing lactate, making LPH attractive. For example, in humans the spinal cord glial cells rely heavily on lactate. Oligodendrocytes, for example, rely on large amounts of lactate for myelination, as it serves as the main precursor for lipogenesis. Sánchez-Abarca et al. (2001) demonstrated that oligodendrocytes used ∼5x as much lactate as either neurons or astrocytes. This reliance on lactate is critical in the human spinal cord, where the glia-neuron ratio is markedly higher than other mammalian spinal cords (∼6–7 vs. ∼1–2), and even higher than that of the cerebral cortex (Bahney and von Bartheld, 2019). Supporting the hypothesis that lactate is critical to spinal cord function, patients with amyotrophic lateral sclerosis (ALS) express lower amounts of MCT1, and non-ALS patients exhibit ALS-type symptoms when MCT1 is inhibited, both presumably due to impairment of oligodendrocyte lactate transport (Rinholm, 2011). Mouse ALS models further support this mechanism, as the ALS mouse is also deficient in MCT1 (Rinholm, 2011).

The “milieu” of the central nervous system has long been known to be a “lactate rich” environment, typically with an extracellular space [lactate] of around 2.5 mM (Turner and Adamson, 2011). In work on a transgenic mouse model of ASPS, the cranial vault (not in any particular type of tissue, but rather in the space of the that environment) proved to be where tumors formed preferentially; administration of exogenous lactate increased this tumorigenesis (Goodwin et al., 2014). Cranial vault lactate concentration proved to be on the order of ≈6–8 mM and increased with daily lactate injections. The human cranial vault, however, has a much lower lactate concentration, and presumably provides a different tumor microenvironment than the mouse (Turner and Adamson, 2011; Goodwin et al., 2014). Does this difference in lactate concentration influence fuel choice by cells? According to work by Sonveaux et al. (2008) and others, some tumor cells may prefer lactate as a fuel, potentially rendering LPH relatively useless. However, as noted earlier, the context in which many of these experiments were performed must be noted, as many used lactate concentrations that were ≈10x the normal concentration. Again, this concentration is known to induce lactate uptake into almost any cell it bathes (Pagliassotti and Donovan, 1990).

## Conclusion and Future Directions

Utilizing an approach that lowers glucose while providing lactate (i.e., “LPH”) is a tantalizing and novel way of targeting tumors, given the reliance of many host tissues on lactate, and the preference of many tumors to produce rather than consume lactate. Many tumors likely differ in not only their preference for lactate, but also in their MCT expression profile in comparison to the non-tumor tissue around it. This may potentially permit selective inhibition of particular MCTs in an effort to target tumor cells. For example, if a tumor presents in the CNS that expresses MCT4 but little MCT1, inhibition of MCT4 may selectively target tumor cells while allowing non-tumor tissue to continue its use of lactate as a fuel. Because MCT1 and 2 are expressed throughout the CNS as noted (e.g., MCT2s in neurons and MCT1s along with MCT4s in astrocytes), inhibition of MCT4 would theoretically stop a tumor’s sole mechanism for transporting lactate, while CNS tissues would have another MCT subtype that could increase its activity accordingly (e.g., MCT1s in astrocytes). In this scenario, MCT4 blockade with or without LPH could prove efficacious in targeting tumors. Currently more experimentation is needed, as (1) tumor cells can exhibit a significant amount of metabolic plasticity (Ždralevic et al., 2017), and (2) there is potential for dramatic deleterious side effects in the CNS when inhibiting lactate transport (Rinholm, 2011). Studies are currently ongoing in our lab examining this and other combinations in various tumor lines.

In conclusion, lactate is the “central” molecule in both whole body and central nervous system metabolism. Many neural tissues utilize lactate, highlighted by shuttles within the brain, but also by the critical role lactate plays as a lipogenic precursor in the human spinal cord. Targeting lactate metabolism in different diseases is a promising possibility in the near future, but care must be taken to understand the normal physiology of lactate in the CNS. Future work will determine the efficacy of targeting central nervous system tumors utilizing an LPH-based approach.

## Data Availability Statement

All datasets generated for this study are included in the article/supplementary material, further inquiries can be directed to the corresponding author.

## Author Contributions

MG, LG, and MN had active roles in developing the concept and experiments described, which has included years of working together and discussing both these results and the underlying concepts. MG wrote the initial draft of the manuscript. LG and MN edited that and subsequent drafts. The entire work was a collaborative effort from all authors.

## Conflict of Interest

MG received royalty on a textbook, received one time payments for consultation for Augmedics and ROM3. None of these are related to this article. The remaining authors declare that the research was conducted in the absence of any commercial or financial relationships that could be construed as a potential conflict of interest.
